# A Brain Signature to Differentiate Acute and Chronic Pain in Rats

**DOI:** 10.3389/fncom.2016.00041

**Published:** 2016-04-28

**Authors:** Yifei Guo, Yuzheng Wang, Yabin Sun, Jin-Yan Wang

**Affiliations:** ^1^Key Laboratory of Mental Health, Institute of Psychology, Chinese Academy of SciencesBeijing, China; ^2^School of Humanities, University of Chinese Academy of SciencesBeijing, China

**Keywords:** acute pain, chronic pain, auditory-evoked potentials (AEPs), sensory processing, animal models

## Abstract

The transition from acute pain to chronic pain entails considerable changes of patients at multiple levels of the nervous system and in psychological states. An accurate differentiation between acute and chronic pain is essential in pain management as it may help optimize analgesic treatments according to the pain state of patients. Given that acute and chronic pain could modulate brain states in different ways and that brain states could greatly shape the neural processing of external inputs, we hypothesized that acute and chronic pain would show differential effects on cortical responses to non-nociceptive sensory information. Here by analyzing auditory-evoked potentials (AEPs) to pure tones in rats with acute or chronic pain, we found opposite influences of acute and chronic pain on cortical responses to auditory inputs. In particular, compared to no-pain controls, the N100 wave of rat AEPs was significantly enhanced in rats with acute pain but significantly reduced in rats with chronic pain, indicating that acute pain facilitated cortical processing of auditory information while chronic pain exerted an inhibitory effect. These findings could be justified by the fact that individuals suffering from acute or chronic pain would have different vigilance states, i.e., the vigilance level to external sensory stimuli would be increased with acute pain, but decreased with chronic pain. Therefore, this auditory response holds promise of being a brain signature to differentiate acute and chronic pain. Instead of investigating the pain system *per se*, the study of pain-induced influences on cortical processing of non-nocicpetive sensory information might represent a potential strategy to monitor the progress of pain chronification in clinical applications.

## Introduction

Acute pain, which serves as a warning signal of injury or illness, normally comes on quickly and lasts for a short time (Carr and Goudas, 1999; Apkarian et al., 2009). If not treated properly, acute pain can develop into chronic pain in which the pain persists even after the initial injury or illness is healed (Merskey and Bogduk, 1994). When this happens, considerable changes occur in both the peripheral and central nervous systems (CNS) as well as in the psychological profiles of individuals (May, 2008). An accurate differentiation between acute and chronic pain is essential in pain management as it may help optimize analgesic treatments according to the pain state of patients (Loeser and Melzack, 1999; Chou and Huffman, 2007a,b). It is, however, very difficult to make such a differentiation during pain chronification, and a commonly-used operational approach for this purpose is purely based on the duration of pain (e.g., pain that lasts for more than 3 or 6 months is defined as chronic pain; Merskey and Bogduk, 1994). This approach can be highly unreliable because it ignores the substantial individual differences in the process of pain chronification (Lavand’homme, 2011). Nor can questionnaires be relied on to distinguish acute pain from chronic pain, since patients may sometimes describe the two pain states with equivalent characteristics (Hashmi et al., 2013).

Some recent studies have found that information about the transition from acute pain to chronic pain could be documented by changes in brain structure and function (May, 2008; Apkarian et al., 2009, 2011), for example, a large-scale reorganization of brain activities towards emotional circuits could occur during the chronification of back pain (Hashmi et al., 2013) and brain structural and functional connectivity may be able to predict that process (Baliki et al., 2012; Mansour et al., 2013). Importantly, the modulated brain structure and function could influence cortical processing of various sensory information—not only nociceptive information (Apkarian et al., 2005; Wiech et al., 2008) but also non-nociceptive information (Gilbert and Sigman, 2007; Fontanini and Katz, 2008). Consistent with this, besides the large number of studies that focused on the functionality of the nociceptive system *per se* in pain states, there are reports of distorted cortical processing of non-nociceptive sensory inputs (e.g., auditory and visual stimuli) in individuals with acute pain (Johnson and Adler, 1993; Lorenz and Bromm, 1997; Bingel et al., 2007) or chronic pain (Lorenz et al., 1997; Wang et al., 1999; Blomhoff et al., 2000; Ambrosini et al., 2003; Carrillo-de-la-Peña et al., 2006; Casale et al., 2008) in experimental settings. Given that acute pain and chronic pain may modulate brain activities in different ways (Apkarian et al., 2009, 2011), we hypothesized that the pain-related distortions of non-nociceptive sensory processing could be differently represented when pain shifts from acute to chronic states. If this hypothesis is valid, it would suggest that examining the pain-related distortions of non-nociceptive sensory processing might be a viable strategy for monitoring pain chronification and thus could be potentially applied in clinical practice.

Here we tested this hypothesis by investigating the different influences of acute pain and chronic pain on auditory-evoked potentials (AEPs) using rat models. An acute inflammatory pain model was produced by intraplantar injection of formalin, and a chronic inflammatory pain model was produced by intraplantar injection of complete Freund’s adjuvant (CFA). In both pain models, multi-channel AEPs elicited by pure tones in freely-moving rats were recorded and compared.

## Materials and Methods

### Animals

Sixty-four male Sprague-Dawley rats (weight at arrival: 180–200 g; Laboratory Animal Center, Academy of Military Medical Sciences, Beijing, China) were used in the experiments. Animals were housed individually under controlled temperature (22 ± 2°C) and humidity (50 ± 10%) conditions with a reversed 12 h light/dark cycle (light on at 7:00 PM). They were handled daily for a week before electrode implantation surgery. All experimental procedures were in accordance with the National Institutes of Health Guide for the Care and Use of Laboratory Animals and approved by the ethics committee of the Institute of Psychology, Chinese Academy of Sciences.

### Electrode Implantation

Animals were anesthetized with sodium pentobarbital (50 mg/kg, i.p.) and then secured on a stereotaxic apparatus (Stoelting, Wood Dale, IL, USA). Twelve recording electrodes (stainless steel screws, 1.0 mm in diameter) were implanted symmetrically on the rat skull over both hemispheres according to the following coordinates: (1) electrodes L1 and R1, 5.0 mm anterior to bregma (5.0 A), ± 1.5 mm lateral to midline (± 1.5 L); (2) electrodes L2 and R2, 3.0 A, ± 1.0 L; (3) electrodes L3 and R3, −1.5 A, ± 2.5 L; (4) electrodes L4 and R4, −4.5 A, ± 1.0 L; (5) electrodes L5 and R5, 0.0 A, ± 4.5 L; and (6) electrodes L6 and R6, −4.5 A, ± 5.0 L. A reference and a ground electrode were placed at the midline, 2.0 and 4.0 mm posterior to lambda, respectively. Insulated wires connected the electrodes to a miniature connector, and the whole assembly was firmly attached to the skull with dental cement. After receiving penicillin (160,000 U, i.p.), animals were allowed at least 1 week to recover from the surgery.

### Auditory Stimuli

Auditory stimuli were generated digitally using custom MATLAB (Mathworks, Natick, MA, USA) scripts, amplified by a power amplifier (A-S300, YAMAHA, Hamamatsu, Japan), and delivered through a loudspeaker (H1189–27TDFC, SEAS, Oslo, Norway) mounted in the ceiling of an anechoic sound-attenuated chamber. Recordings were carried out in a Plexiglas cage (L: 23 cm, W: 22 cm, H: 36 cm) situated in the sound-attenuated chamber. The loudspeaker was approximately 1 m from the middle of the test cage. The acoustic system was calibrated with a condenser microphone (C01U, Samson, Hauppauge, NY, USA) and a sound level meter (1350A, TES, Taipei, Taiwan) before the experiments.

AEPs were elicited by pure tones of either 8000 or 8800 Hz presented at 75 dB SPL with 100 ms duration and 500 ms inter-stimulus interval (auditory oddball paradigm). In accordance with previous studies recording auditory responses of the rat brain (Shinba, 1997; Lazar and Metherate, 2003; Jung et al., 2013; Witten et al., 2014), auditory stimuli with higher frequency than those commonly used in human AEP studies (Sambeth et al., 2003) were used in the present study, since rats exhibit more robust electrophysiological responses to higher pitch tones (with a maximum between 8 and 20 KHz) than to lower pitch tones (Knight et al., 1985). Each recording session contained eight stimulation blocks presented in random order with an approximately 1 min break between successive blocks. In four of these blocks, the lower frequency tone served as the standard (i.e., frequent stimuli, 85%) and the higher frequency tone as the deviant (i.e., rare stimuli, 15%). In the other four blocks, the roles of the lower and higher frequency tones were switched. In each block, 260 tones were presented in a pseudorandom order with the constraint that at least two standards were delivered before each deviant. The first 10 stimuli in each block were excluded from off-line analysis in order to minimize the potential influence of switching between different types of blocks on the measured auditory responses (Nakamura et al., 2011). Each block lasted about 2.5 min and an entire session took less than 30 min.

### EEG Recording

Rats were individually placed in the test cage 15–20 min before EEG data collection to familiarize them with the test environment. For EEG recording, a headstage was attached to the connector mounted on the rat’s head and connected to an EEG amplifier (UEA-16BZ, SYMTOP, Beijing, China) via a flexible multi-strand cable. EEG signals were recorded continuously from the 12 recording electrodes, sampled at 1000 Hz, and low-pass filtered at 120 Hz. Rats were allowed to move freely in the test cage throughout the recording session.

### Experimental Procedures

#### Acute Pain Model

Forty rats were randomly divided into four groups: 1% formalin (*n* = 11), 5% formalin (*n* = 10), normal saline (NS) control (*n* = 10), and no-treatment (NT) control (*n* = 9) group. After the rats were placed in the test cage for approximately 20 min, they were injected with 1% formalin, 5% formalin, or NS (50 μL each) subcutaneously into the plantar surface of their left hindpaw according to the group they belonged to. Immediately after injection, the rats were returned to the test cage. Nociceptive behaviors were video-recorded over the following 60 min and quantified by measuring the time spent licking the injected paw within each 5 min period. Rats in the NT group were treated by the same operations but without any injection. AEPs were repeatedly recorded 24 h before (baseline), 20–50 min, 90–120 min, and 24 h after injection. One rat in the 5% formalin group did not show any nociceptive behavior following injection and thus was excluded from further analyses.

#### Chronic Pain Model

Twenty-four rats were randomly divided into two groups: CFA group (*n* = 12) and NS group (*n* = 12). The rats were subcutaneously injected with either 100 μL CFA (Sigma-Aldrich, St. Louis, MO, USA) or NS into the plantar surface of their right hindpaw. AEPs were repeatedly recorded 1 day before (baseline), 1, 3, 7, 14, and 28 days after injection. Thermal nociceptive thresholds, quantified using the paw withdrawal latencies (PWLs) to radiant heat, of the injected and non-injected hindpaws were measured on each test day (PWL test started at least 2 h after the end of EEG recording).

The thermal nociceptive threshold test was adapted from Hargreaves et al. (1988). Rats were placed individually in Plexiglas chambers on an elevated glass floor and habituated to the test apparatus for at least 20 min. Focused radiant heat generated by a 100 W projector lamp was applied through the glass floor to the plantar surface of the stimulated hindpaw. PWL was defined as the time from the onset of heat stimulation to the withdrawal of the hindpaw. A cut-off time of 22 s was employed to avoid tissue damage. Five trials separated by at least 5 min were conducted on each hindpaw. To ensure that the rats were familiarized to the stimulation procedure and to increase the reliability of the measurement, latency of the first trial was discarded, and latencies of the following four trials were averaged to give a mean PWL.

Three rats (one in the CFA group, two in the NS group) did not show any movement of the stimulated hindpaws during the 22 s test period and thus were excluded from further analyses.

### EEG Data Analysis

EEG data were preprocessed using EEGLAB (Delorme and Makeig, 2004), an open source toolbox running in the MATLAB environment, and custom MATLAB scripts. Continuous EEG signals were band-pass filtered between 1 and 30 Hz and segmented into epochs extending from −50 ms to +350 ms relative to the stimulus onset. EEG segments were baseline-corrected using the pre-stimulus interval, and trials contaminated by gross artifacts were manually rejected by visual inspection. Since we aimed to assess the influence of pain states (acute or chronic pain) on AEPs, our analysis was focused on standard-related cortical response due to its higher signal-to-noise ratio than deviant-related cortical response (the number of trials of standard was much larger than that of deviant). For each group, single-trial responses to standard stimuli were averaged for each rat and session. Single-rat average waveforms were subsequently averaged to obtain group-level waveforms for each session. Three distinct components in AEPs were identified, which consisted of an initial negative deflection peaking at ~40 ms after stimulus onset (N40), followed by a positive deflection peaking at ~60 ms (P60) and another negative deflection peaking at ~100 ms (N100). For each group, peak latency and baseline-to-peak amplitude of each component were measured for each rat and session from the electrodes where the deflection reached its maximum. Grand-average scalp topographies at their peak latencies were plotted using a rat head model according to the rat brain atlas of Paxinos and Watson (2007).

### Statistical Analysis

Data are expressed as mean ± standard error (SE). Statistical analyses were performed with STATISTICA 10 (StatSoft, Tulsa, OK, USA) and GraphPad Prism 5.0 (GraphPad Software, La Jolla, CA, USA). Statistical significance was set as *p* < 0.05.

For acute pain model, the licking time was compared using a two-way analysis of variance (ANOVA) with group (three levels: NS, 1% and 5% formalin) as a between-subject factor and time (12 levels: every 5 min during the first hour following injection) as a within-subject factor. The cumulative licking time within the 20–50 min interval after injection was compared among the three injected groups using a one-way ANOVA. For each AEP component, peak latencies were compared using a two-way ANOVA with group (four levels: NT, NS, 1% and 5% formalin) as a between-subject factor and time (four levels: baseline, 20–50 min, 90–120 min, and 24 h after injection) as a within-subject factor. Baseline-to-peak amplitudes of each AEP component were normalized for each subject by dividing the value in each session by the value in the baseline session, and the normalized amplitudes of each AEP component were compared using a two-way ANOVA with group (four levels: NT, NS, 1% and 5% formalin) as a between-subject factor and time (three levels: 20–50 min, 90–120 min, and 24 h after injection) as a within-subject factor. Note that the baseline data were not included in this analysis since in the baseline session the normalized amplitudes, all of which were 1, had no variance. Fisher’s protected least significant difference test was used for *post hoc* comparisons.

For chronic pain model, the paw withdrawal latencies to radiant heat were compared using a three-way ANOVA with group (two levels: NS and CFA) as a between-subject factor, and time (six levels: baseline, 1, 3, 7, 14, and 28 days after injection) and stimulation site (two levels: left and right hindpaws) as within-subject factors. Fisher’s protected least significant difference test was used for *post hoc* comparisons. For each AEP component, peak latencies were compared using a two-way ANOVA with group (two levels: NS and CFA) as a between-subject factor and time (six levels: baseline, 1, 3, 7, 14, and 28 days after injection) as a within-subject factor. Consistent with the analysis for the acute pain model, baseline-to-peak amplitudes of each AEP component were normalized for each subject, and the normalized amplitudes of each AEP component were compared using a two-way ANOVA with group (two levels: NS and CFA) as a between-subject factor and time (five levels: 1, 3, 7, 14, and 28 days after injection) as a within-subject factor. Fisher’s protected least significant difference test was used for *post hoc* comparisons.

## Results

### The Influence of Acute Pain on AEPs

**Figure 1:**
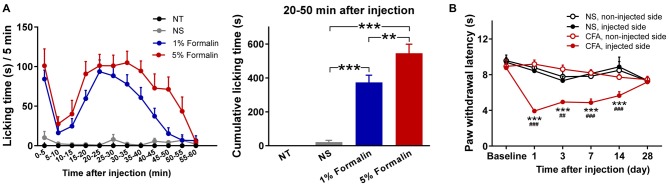
**Nociceptive behaviors of rats in acute and chronic pain models. (A)** Formalin-induced acute pain behaviors. Left: Time spent licking the injected hindpaws within each 5 min period (from 0 to 60 min following the injection). Rats injected with 1% or 5% formalin showed a typical biphasic pattern of licking behavior (phase I: 0 to 5 min; phase II: 15 to 60 min), which was not observed in the NS and NT groups. Right: The cumulative licking time within the 20-50 min interval after injection. During this time interval, rats in the 1% and 5% formalin groups spent significantly longer time to lick their injected paws than rats in the NS group. Rats in the 5% formalin group also showed significantly longer licking time than rats in the 1% formalin group. NT: no treatment; NS, normal saline. **p < 0.01; ***p < 0.001. NT: *n* = 9; NS: *n* = 10; 1% formalin: *n* = 11; 5% formalin: *n* = 9. **(B)** Complete Freund’s adjuvant (CFA)-induced chronic thermal hyperalgesia. Before injection (Baseline), paw withdrawal latency (PWL) to radiant heat stimuli was not significantly different between the NS and CFA groups, nor between the left and right hindpaws. From day 1 to day 14 after injection, PWLs of the injected hindpaw were significantly decreased in the CFA group compared to the NS group. Moreover, in the CFA group, PWLs of the injected hindpaw were significantly decreased compared to those of the non-injected hindpaw. NS, normal saline; CFA, complete Freund’s adjuvant. For the comparison between CFA and NS groups of the injected hindpaw, ^##^*p* < 0.01, ^###^*p* < 0.001. For the comparison between injected and non-injected hindpaws in the CFA group, ***p < 0.001. NS: *n* = 10; CFA: *n* = 11. Data are expressed as mean ± standard error (SE).

Nociceptive behaviors, quantified by measuring the time spent licking the injected paw within each 5 min period, are summarized in Figure 1A (left). Rats injected with 1% or 5% formalin, but not those injected with NS, exhibited a typical biphasic pattern of licking behavior (phase I: 0–5 min; phase II: 15–60 min). This observation is consistent with that of previous reports on the temporal profile of formalin-induced acute pain (licking behaviors usually subside within 1 h, while some other spontaneous nociceptive behaviors may last up to approximately 2 h; Dubuisson and Dennis, 1977; Porro and Cavazzuti, 1993), which justifies the validity of the acute pain model. Two-way ANOVA revealed that the licking time was significantly modulated by “group” (*F*_(2,27)_ = 38.4, *p* < 0.0001), “time” (*F*_(11,297)_ = 16.7, *p* < 0.0001), and their interaction (*F*_(22,297)_ = 4.8, *p* < 0.0001). The cumulative licking time within the 20–50 min interval after injection was significantly different among the three injected groups (*F*_(2,27)_ = 45.3, *p* < 0.0001, one-way ANOVA; Figure 1A, right). *Post hoc* comparisons revealed that the cumulative licking time was significantly different between each pair of the injected groups (NS vs. 1% formalin: *p* < 0.001; NS vs. 5% formalin: *p* < 0.001; 1% formalin vs. 5% formalin: *p* < 0.01).

**Figure 2:**
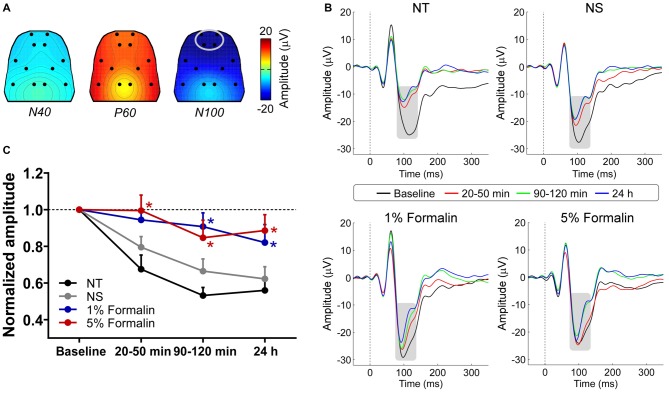
**The influence of acute pain on AEPs. (A)** Grand-average scalp topographies of N40, P60, and N100 waves. **(B)** For each group, AEP waveforms from different sessions are plotted in different colors and superimposed. Displayed waveforms were measured from fronto-central electrodes (L1, R1, L2, and R2; enclosed by the light gray ellipse in **(A)**, where the N100 wave (marked using gray rectangles) displayed a negative maximum. **(C)** After injection, the normalized N100 amplitudes in the 1% and 5% formalin groups were significantly larger than those in the NS and NT groups for any post-injection session. NT: no treatment; NS, normal saline. *p < 0.05, compared to the NS group. NT: *n* = 9; NS: *n* = 10; 1% formalin: *n* = 11; 5% formalin: *n* = 9. Data are expressed as mean ± SE.

The group-level average AEP waveforms were characterized by three distinct components: N40, P60, and N100. Whereas the N40 and P60 waves were maximal over the frontal and bilateral temporal regions respectively, the N100 wave displayed a negative maximum over the fronto-central area (Figure 2A). Therefore, in the subsequent analyses, peak latencies and amplitudes of these waves were measured from the waveforms averaged across the following electrodes: L1 and R1 for N40; L6 and R6 for P60; L1, R1, L2, and R2 for N100 (Figure 2B).

Peak latencies and amplitudes of N40, P60, and N100 for different groups and sessions are summarized in Table 1. Two-way ANOVA revealed that peak latencies of N40 were not significantly modulated by “group”, “time”, or their interaction (detailed statistics are summarized in Table 2). Peak latencies of P60 and N100 were only significantly modulated by “time” (P60: *F*_(3, 105)_ = 8.1, *p* < 0.0001; N100: *F*_(3,105)_ = 25.1, *p* < 0.0001). Normalized N40 amplitudes were not significantly modulated by “group”, “time”, or their interaction. Normalized P60 amplitudes were only significantly modulated by “time” (*F*_(2,70)_ = 4.4, *p* = 0.016). In contrast, normalized N100 amplitudes were significantly modulated by “group” (*F*_(3,35)_ = 6.2, *p* = 0.002) and “time” (*F*_(2,70)_ = 8.1; *p* = 0.0007), but not by their interaction (*F*_(6,70)_ = 0.5; *p* = 0.81; Figure 2C). *Post hoc* comparisons revealed that normalized N100 amplitudes in the 1% and 5% formalin groups were significantly larger than those in the NS and NT groups for any post-injection session (*p* < 0.05 for all comparisons except for the marginal significance (*p* = 0.06) between 1% formalin and NS groups during the 20–50 min interval after injection). Normalized N100 amplitudes were not significantly different between NS and NT groups, as well as between 1% and 5% formalin groups for any post-injection session (*p* > 0.05 for all comparisons).

**Table 1:** **Latency and amplitude of AEP components for different groups and sessions (acute pain model)**.

	Latency (ms)			Amplitude (μV)		
	N40	P60	N100	N40	P60	N100
NT group (*n* = 9)
Baseline	38.3 ± 0.9	61.9 ± 0.6	112.0 ± 3.5	−8.1 ± 1.4	17.7 ± 2.9	−26.3 ± 4.8
20–50 min	38.2 ± 0.7	61.2 ± 0.6	101.9 ± 2.2	−6.3 ± 0.9	12.5 ± 1.6	−16.7 ± 3.8
90–120 min	38.8 ± 0.6	61.6 ± 0.5	102.3 ± 2.9	−6.5 ± 0.9	13.5 ± 1.2	−13.3 ± 2.7
24 h	38.6 ± 1.0	61.0 ± 0.6	99.3 ± 2.5	−7.0 ± 0.8	12.7 ± 1.9	−14.3 ± 3.3
NS group (*n* = 10)
Baseline	39.4 ± 0.4	60.6 ± 0.5	105.1 ± 2.6	−7.5 ± 0.6	12.4 ± 1.1	−29.1 ± 5.3
20–50 min	39.6 ± 0.4	60.5 ± 0.6	98.5 ± 1.8	−7.1 ± 0.9	12.3 ± 1.5	−22.9 ± 4.3
90–120 min	39.5 ± 0.3	60.4 ± 0.7	97.4 ± 2.1	−7.8 ± 0.7	12.1 ± 1.5	−20.3 ± 5.4
24 h	39.2 ± 0.3	60.6 ± 0.7	96.1 ± 1.8	−7.1 ± 0.7	11.5 ± 1.1	−20.4 ± 5.6
1% Formalin group (*n* = 11)
Baseline	38.7 ± 0.4	60.9 ± 0.7	102.2 ± 3.1	−6.9 ± 0.7	21.1 ± 2.9	−29.8 ± 5.4
20–50 min	38.5 ± 0.7	59.4 ± 0.7	97.7 ± 1.9	−6.3 ± 0.8	14.9 ± 2.0	−27.2 ± 4.0
90–120 min	38.4 ± 0.4	60.4 ± 0.5	95.1 ± 1.3	−7.1 ± 0.8	20.0 ± 2.8	−26.5 ± 4.9
24 h	38.6 ± 0.5	60.5 ± 0.7	91.7 ± 1.0	−6.8 ± 0.9	17.0 ± 2.4	−24.1 ± 4.7
5% Formalin group (*n* = 9)
Baseline	38.3 ± 0.6	60.6 ± 0.5	103.2 ± 4.3	−7.6 ± 1.0	15.0 ± 1.8	−25.8 ± 5.1
20–50 min	37.8 ± 0.7	58.9 ± 0.5	98.0 ± 1.9	−5.8 ± 0.7	11.9 ± 1.1	−25.4 ± 3.7
90–120 min	38.2 ± 0.7	60.1 ± 0.3	95.6 ± 1.7	−5.2 ± 1.3	15.1 ± 2.2	−22.6 ± 4.9
24 h	38.3 ± 0.7	60.2 ± 0.6	97.3 ± 3.9	−6.9 ± 1.0	15.0 ± 1.9	−24.1 ± 6.0

*Data are expressed as mean ± SE. NT, no treatment; NS, normal saline*.

**Table 2:** **Two-way ANOVA exploring the effect of “group” and “time” on latency and amplitude of AEP components (acute pain model)**.

	Latency						Normalized amplitude					
	N40		P60		N100		N40		P60		N100	
	*F*	*p*	*F*	*p*	*F*	*p*	*F*	*p*	*F*	*p*	*F*	*p*
Group	1.0	0.43	1.3	0.30	2.2	0.10	0.8	0.49	2.1	0.12	6.2	**0.002**
Time	0.5	0.70	8.1	**<0.0001**	25.1	**<0.0001**	0.5	0.61	4.4	**0.02**	8.1	**0.0007**
Interaction	0.7	0.71	1.8	0.08	0.8	0.60	1.0	0.41	2.0	0.08	0.5	0.81

*p values in boldface indicate statistically significant results*.

These results demonstrated that N100 amplitude of AEPs was significantly enhanced in rats with acute pain (1% and 5% formalin groups) compared to control rats (NT and NS groups), which indicated that acute pain would facilitate the cortical processing of auditory information in rats. Such facilitation effect existed not only when formalin-injected rats exhibited robust nociceptive behaviors but also when the apparent nociceptive behaviors had subsided, e.g., 24 h after formalin injection.

### The Influence of Chronic Pain on AEPs

Nociceptive thresholds, quantified by measuring PWLs to radiant heat of the injected and non-injected hindpaws, are summarized in Figure 1B. Rats injected with CFA exhibited pronounced thermal hyperalgesia that developed within 1 day and persisted through 14 days following injection. This observation is similar to that of previous reports on the temporal profile of thermal hyperalgesia in CFA-induced chronic pain model (Wang et al., 2009; Li et al., 2012). Three-way ANOVA revealed that PWLs were significantly modulated by “group” (*F*_(1,19)_ = 5.8, *p* = 0.03), “time” (*F*_(5,95)_ = 7.5, *p* < 0.0001), “stimulation site” (*F*_(1,19)_ = 43.8, *p* < 0.0001), interactions between two factors (“group” × “stimulation site”: *F*_(1,19)_ = 41.0, *p* < 0.0001 ; “time” × “stimulation site”: *F*_(5,95)_ = 9.5, *p* < 0.0001; “group” × “time”: marginal significance, *F*_(5,95)_ = 2.2, *p* = 0.06), and the interaction between three factors (*F*_(5,95)_ = 7.6, *p* < 0.0001). *Post hoc* comparisons revealed that PWLs of the injected hindpaw were significantly shorter in the CFA group than in the NS group (*p* < 0.01 for all comparisons 1, 3, 7, and 14 days after injection). In the CFA group, PWLs of the injected hindpaw were significantly shorter than those of the non-injected hindpaw (*p* < 0.001 for all comparisons 1, 3, 7, and 14 days after injection).

**Figure 3:**
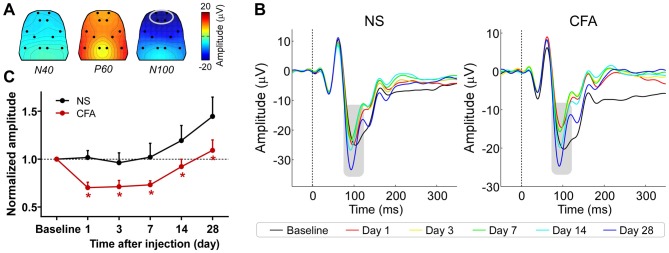
**The influence of chronic pain on AEPs. (A)** Grand-average scalp topographies of N40, P60, and N100 waves. **(B)** For each group, AEP waveforms from different sessions are plotted in different colors and superimposed. Displayed waveforms were measured from fronto-central electrodes (L1, R1, L2, and R2; enclosed by the light gray ellipse in **(A)**, where the N100 wave (marked using gray rectangles) displayed a negative maximum. **(C)** After injection, the normalized N100 amplitudes in the CFA group were significantly smaller than those in the NS group for any post-injection session. NS, normal saline; CFA, complete Freund’s adjuvant. *p < 0.05, compared to the NS group. NS: *n* = 10; CFA: *n* = 11. Data are expressed as mean ± SE.

The group-level average AEPs of the chronic pain rats consisted of three distinct components (N40, P60, and N100), whose polarity and order were markedly similar to AEPs of the acute pain rats (Figures 3A,B). A comparison between Figures 2A, 3A revealed high consistency in scalp topographies of the AEPs between the acute and chronic pain conditions, indicating that changes in pain state may not alter the spatial features of the auditory evoked cortical responses.

Peak latencies and amplitudes of N40, P60, and N100 for different groups and sessions are summarized in Table 3. Two-way ANOVA revealed that peak latencies of N40 and N100 were only significantly modulated by “time” (N40: *F*_(5,95)_ = 3.1, *p* = 0.01; N100: *F*_(5,95)_ = 26.2, *p* < 0.0001; Table 4). Peak latencies of P60 were not significantly modulated by “group”, “time”, or their interaction (Table 4). Normalized N40 and P60 amplitudes were not significantly modulated by “group”, “time”, or their interaction. In contrast, normalized N100 amplitudes were significantly modulated by “group” (*F*_(1,19)_ = 5.0, *p* = 0.038) and “time” (*F*_(4,76)_ = 15.4; *p* < 0.0001), but not by their interaction (*F*_(4,76)_ = 0.2; *p* = 0.95; Figure 3C). *Post hoc* comparisons revealed that normalized N100 amplitudes in the CFA group were significantly reduced compared to those in the NS group for any post-injection session (*p* < 0.05 for all comparisons).

**Table 3:** **Latency and amplitude of AEP components for different groups and sessions (chronic pain model)**.

	Latency (ms)			Amplitude (μV)		
	N40	P60	N100	N40	P60	N100
NS group (*n* = 10)
Baseline	39.0 ± 0.3	59.8 ± 0.5	105.9 ± 3.0	−7.9 ± 0.6	13.6 ± 1.5	−26.7 ± 4.2
Day 1	39.2 ± 0.3	59.9 ± 0.5	100.6 ± 2.8	−7.8 ± 0.5	12.7 ± 1.0	−25.8 ± 3.7
Day 3	39.5 ± 0.5	60.4 ± 0.5	96.7 ± 1.5	−7.3 ± 0.7	14.7 ± 1.2	−24.3 ± 3.6
Day 7	39.0 ± 0.3	59.3 ± 0.4	95.1 ± 1.7	−7.6 ± 0.6	14.2 ± 1.5	−24.2 ± 3.0
Day 14	38.2 ± 0.5	59.5 ± 0.4	92.2 ± 1.1	−7.5 ± 0.6	14.3 ± 1.8	−27.5 ± 2.9
Day 28	38.8 ± 0.4	60.0 ± 0.3	93.9 ± 1.4	−7.8 ± 0.6	16.7 ± 1.9	−34.6 ± 4.6
CFA group (*n* = 11)
Baseline	39.0 ± 0.5	60.0 ± 0.6	109.7 ± 4.3	−7.8 ± 0.7	9.7 ± 1.6	−22.0 ± 2.5
Day 1	38.9 ± 0.4	59.7 ± 0.6	99.3 ± 2.4	−6.1 ± 0.9	11.1 ± 1.4	−16.0 ± 2.6
Day 3	39.1 ± 0.5	60.5 ± 0.6	94.5 ± 1.9	−6.0 ± 0.8	11.2 ± 1.4	−16.3 ± 2.7
Day 7	38.6 ± 0.4	60.7 ± 0.4	95.5 ± 1.8	−6.3 ± 0.9	12.7 ± 2.2	−16.6 ± 2.3
Day 14	38.4 ± 0.5	60.7 ± 0.6	94.4 ± 1.4	−6.1 ± 0.4	12.3 ± 1.6	−20.5 ± 2.7
Day 28	38.9 ± 0.5	60.4 ± 0.6	93.0 ± 1.6	−6.2 ± 0.7	12.2 ± 1.8	−25.4 ± 4.3

*Data are expressed as mean ± SE. NS, normal saline; CFA, complete Freund’s adjuvant*.

**Table 4:** **Two-way ANOVA exploring the effect of “group” and “time” on latency and amplitude of AEP components (chronic pain model)**.

	Latency						Normalized amplitude					
	N40		P60		N100		N40		P60		N100	
	*F*	*p*	*F*	*p*	*F*	*p*	*F*	*p*	*F*	*p*	*F*	*p*
Group	0.07	0.80	0.9	0.38	0.02	0.90	3.5	0.08	1.4	0.26	5.0	**0.038**
Time	3.1	**0.01**	1.1	0.39	26.20	**<0.0001**	0.5	0.75	1.3	0.29	15.40	**<0.0001**
Interaction	0.4	0.83	2.0	0.09	1.1	0.36	0.2	0.96	0.6	0.65	0.2	0.95

*p values in boldface indicate statistically significant results*.

These results showed that N100 amplitude of AEPs was significantly reduced in rats with chronic pain (CFA group) compared to control rats (NS group), which indicated that chronic pain would inhibit the cortical processing of auditory information in rats. This inhibitory effect persisted throughout the observation period of 28 days.

## Discussion

We observed opposite influences of acute and chronic pain on cortical responses to auditory inputs using rat models. On one hand, N100 wave of rat AEPs was significantly enhanced in rats with acute pain compared to no-pain controls, suggesting that acute pain facilitated cortical processing of auditory information. On the other hand, N100 wave of rat AEPs was significantly reduced in rats with chronic pain compared to no-pain controls, suggesting that chronic pain inhibited cortical processing of auditory information. Our observations could not be explained by the direct interaction between nociceptive and non-nociceptive sensory inputs, since such interaction could not yield the opposite effects of acute and chronic pain. Instead, our observations could be justified by the fact that individuals who are suffering from acute or chronic pain would have different vigilance states, i.e., the level of vigilance to external sensory stimuli would be increased with acute pain, but decreased with chronic pain. Since the neural processing of auditory information was biased by acute and chronic pain in opposite directions, AEPs might be used as a representative brain response to distinguish acute pain from chronic pain and to monitor the progress of pain chronification.

### Acute Pain Facilitates Cortical Processing of Auditory Information

Pain, in its acute state, serves as a warning signal of tissue damage and induces protective responses that facilitate recuperation (Woolf, 1995; Millan, 1999; Milligan and Watkins, 2009). The presence of acute pain can result in a remarkably heightened level of general arousal and vigilance of the suffered individual (Millan, 1999; Price, 2000), which could be reflected by the increased attention to potential threats or dangers in the environment (Oken et al., 2006). Note that the increased attention to external changes would be important as it allows the suffered individual to respond properly in life-threatening situations.

Here, we observed a significant enhancement of cortical response to auditory stimuli in rats experiencing acute pain compared to no-pain controls (Figure 2C), which indicated that acute pain could facilitate brain responses to external sensory inputs likely through triggering a surge in vigilance. Consistently, as demonstrated in some human brain imaging studies (Peyron et al., 1999, 2000), activations of bilateral thalamus and upper brainstem in response to acute pain were assumed to partly reflect a generalized arousal enhancement. In addition, neural processing of sensory inputs is highly susceptible to fluctuations in vigilance/arousal (Mackworth, 1968; Davis and Whalen, 2001; Oken et al., 2006), demonstrating an enhanced processing at an increased vigilance level (van Marle et al., 2009; Shackman et al., 2011). All these lines of evidence justify the significant influence of acute pain on brain state (i.e., increased vigilance level/attending to external changes), which would subsequently enhance the cortical processing of non-nociceptive sensory information.

Even though we have provided evidence showing that acute pain could influence the brain state (i.e., the vigilance level) significantly, we believe that their relationship is not straightforward. First, we showed that the facilitatory effect of acute pain could be sustained even when the prominent nociceptive behaviors had subsided. This observation would indicate the dissociation between acute pain and brain state (represented by the facilitatory effect) in the perspective of *duration*. Second, although the 5% formalin group showed clearly more intense nociceptive behaviors (Figure 1A), the normalized N100 amplitudes of AEPs were not significantly different between rats injected with 1% formalin and those injected with 5% formalin (Figure 2C). This observation would demonstrate the dissociation between acute pain and brain state in the perspective of *intensity*. Indeed, the detailed relationship between acute pain and brain state (or the facilitatory effect) should be investigated in the future.

### Chronic Pain Inhibits Cortical Processing of Auditory Information

It is well documented that sleep disturbance and fatigue, consequent to the suffering of chronic pain, are of the most common complaints among chronic pain patients (Ashburn and Staats, 1999; Hart et al., 2000; Smith and Haythornthwaite, 2004). As demonstrated by electrophysiological activities and/or vigilance-related cognitive performance (Belyavin and Wright, 1987; Cajochen et al., 1995, 1999; Cote et al., 2003; Ziino and Ponsford, 2006; Lim and Dinges, 2008), both factors would considerably reduce one’s level of vigilance. Following, the attenuated level of vigilance (modulated brain state) could affect the brain responses to sensory inputs (Fruhstor and Bergström, 1969; Corsi-Cabrera et al., 1999; Cote et al., 2003). Moreover, in contrast to acute pain that would increase the individuals’ attention to potential threats or dangers in the environment, chronic pain would lead to excessive attention to the internal changes (e.g., hypervigilance to pain and other somatic signals (Eccleston and Crombez, 1999, 2007; Crombez et al., 2005)) in patients. The focus on internal changes in chronic pain state would also result in a decreased level of vigilance to the external environment, and thus lead to a detrimental effect on the processing of pain-irrelevant, external signals.

Here, we observed significant attenuation of cortical response to auditory stimuli in rats with chronic pain compared to no-pain controls (Figure 3C). Note that our observation is consistent with some previous reports of sensory impairments in chronic pain patients (Evers et al., 1997; Lorenz et al., 1997; Buodo et al., 2004; Firat et al., 2006; Veldhuijzen et al., 2006; Casale et al., 2008; Korostenskaja et al., 2011). In addition, relevant phenomena have been found in rat models of chronic pain with either inflammatory (Millecamps et al., 2004) or neuropathic (Low et al., 2012) origin, which showed that rats in chronic pain state exhibited decreased ability to perceive small changes in the environment. All these evidences demonstrated that chronic pain could greatly influence the brain state (i.e., decreased vigilance level to external changes), which subsequently attenuated the cortical processing of non-nociceptive sensory information.

Similar to the relationship between acute pain and brain state, the relationship between chronic pain and brain state is also not straightforward. Although we did not assess the influence of chronic pain on brain state in the perspective of *intensity* in the present study, we showed that the inhibitory effect of chronic pain on auditory processing still existed on day 28 (Figure 3C) when the thermal hyperalgesia was abolished (Figure 1B). This observation would suggest the dissociation between chronic pain and brain state (represented by the inhibitory effect) in the perspective of *duration*. Note that this dissociation would be crucial as it implied that the treatment of chronic pain should not only aim to relieve patients from pain, but also be designed to eliminate possible co-morbidities of chronic pain (e.g., alterations in brain state), especially considering that some of the co-morbidities could persist even when the chronic pain has been released (Chapman and Dunbar, 1998).

Although we found that the inhibitory effect of chronic pain could be reliably observed throughout our observation period of 28 days, we have also noticed an increase in N100 amplitude on day 14 and day 28 compared to those in the previous sessions in both the pain and no-pain groups (Figure 3C). We conjecture that the pronounced restoration or enhancement of N100 amplitude in the last two sessions was due most likely to the prolonged inter-session intervals (1 or 2 weeks) from day 7 to day 28 in contrast to the 2- or 4-day intervals for the previous sessions, which is consistent with the dishabituation effect on event-related potentials after longer intervals during repeated tests as reported previously (Kinoshita et al., 1996).

### Transition of Brain States During Pain Chronification

The transition from acute pain to chronic pain has been proven to involve large-scale reorganizations of brain functions (Baliki et al., 2006; Geha et al., 2007; Malinen et al., 2010; Farmer et al., 2011; Parks et al., 2011; Weissman-Fogel et al., 2011). In general, whereas acute pain largely activates brain regions involved in nociceptive information processing (Apkarian et al., 2005), chronic pain is consistently and substantially encoded by brain regions related to emotional and motivational states of patients (Apkarian et al., 2011). A recent longitudinal study illuminated how such a change in brain activation pattern emerged during pain chronification in a group of patients with subacute back pain (Hashmi et al., 2013). It showed that the brain representation for the perception of back pain underwent large-scale reorganization from nociceptive processing regions (including insula, thalamus and anterior cingulate cortex) to emotional relevant circuits (including medial prefrontal cortex and amygdala) as the pain transitioned from subacute state into persistence over a 1-year period (Hashmi et al., 2013). This finding was confirmed by the results of an inter-subject comparison between acute/subacute and chronic back pain patients (Hashmi et al., 2013), as well as the results obtained from other cross-sectional analyses (Apkarian et al., 2005; Baliki et al., 2006, 2010).

Apkarian et al. (2005) pointed out that the increased engagement of cognitive/emotional circuits in chronic pain conditions indicated that chronic pain is different from acute pain in terms of the cognitive, emotional, and introspective components of pain. They expounded this notion in later works, suggesting that a transition in the salience of pain—from viewing a pain perception as an index of external threat to a representation of an internal disease state—is involved in the transition from acute to chronic pain (Apkarian et al., 2009), and may be sufficient to drive the shift in brain representations of pain perception from acute to chronic conditions (Hashmi et al., 2013). Therefore, acute and chronic pain should not be simply described by different duration of pain, but actually represent two distinct states of the system. Our observation that acute pain enhanced the neural processing of auditory information while chronic pain suppressed it would represent such transition of brain states. For this reason, the AEPs, as a representative brain response to monitor the efficiency of the system to process external sensory inputs, may be potentially used to differentiate the brain states related to acute and chronic pain.

### Limitations and Future Directions

We investigated the influences of acute and chronic pain on neural responses to auditory inputs using rat models. Indeed, these influences were observed at limited time points, which hampered us to continually monitor the progress of pain chronification. A longitudinal study that encompasses acute and chronic pain stages, as well as the critical period within which the acute-chronic transition occurs, would be necessary in the future to provide a fine-grained temporal profile of how the brain response changes during pain chronification. In addition, the sensitivity and specificity of using non-nociceptive brain response, e.g., AEPs, to discriminate between acute and chronic pain states should be characterized before using this response to monitor the progress of pain chronification. Importantly, even though animal models have been used to improve our understanding of pain mechanisms, we are aware that the information obtained from animal models cannot be directly applied to humans, and our findings should be replicated in human pain conditions for potential use in the clinic.

## Author Contributions

YG, YW, YS, and J-YW designed the research; YG, YW, and YS performed the research; YG, YW, and J-YW analyzed the data; and YG and JW wrote the article.

## Funding

J-YW is supported by the National Natural Science Foundation of China (NSFC: 31271092) and the Youth Innovation Promotion Association of the Chinese Academy of Sciences.

## Conflict of Interest Statement

The authors declare that the research was conducted in the absence of any commercial or financial relationships that could be construed as a potential conflict of interest.
